# Motorcycle-related hospitalization of adolescents in a Level I trauma center in southern Taiwan: a cross-sectional study

**DOI:** 10.1186/s12887-015-0419-3

**Published:** 2015-08-28

**Authors:** Chi-Cheng Liang, Hang-Tsung Liu, Cheng-Shyuan Rau, Shiun-Yuan Hsu, Hsiao-Yun Hsieh, Ching-Hua Hsieh

**Affiliations:** Department of Trauma Surgery, Kaohsiung Chang Gung Memorial Hospital and Chang Gung University College of Medicine, No.123, Ta-Pei Road, Niao-Sung District, Kaohsiung City, 833 Taiwan; Department of Neurosurgery, Kaohsiung Chang Gung Memorial Hospital and Chang Gung University College of Medicine, Kaohsiung City, Taiwan

**Keywords:** Abbreviated injury scale, Adolescent, Glasgow coma scale, Injury severity score, New injury severity score, Motorcycle, Trauma, Trauma injury severity score

## Abstract

**Background:**

The aim of this study was to investigate and compare the injury pattern, mechanisms, severity, and mortality of adolescents and adults hospitalized for treatment of trauma following motorcycle accidents in a Level I trauma center.

**Methods:**

Detailed data regarding patients aged 13–19 years (adolescents) and aged 30–50 years (adults) who had sustained trauma due to a motorcycle accident were retrieved from the Trauma Registry System between January 1, 2009 and December 31, 2012. The Pearson’s chi-squared test, Fisher’s exact test, or the independent Student’s t-test were performed to compare the adolescent and adult motorcyclists and to compare the motorcycle drivers and motorcycle pillion.

**Results:**

Analysis of Abbreviated Injury Scale (AIS) scores revealed that the adolescent patients had sustained higher rates of facial, abdominal, and hepatic injury and of cranial, mandibular, and femoral fracture but lower rates of thorax and extremity injury; hemothorax; and rib, scapular, clavicle, and humeral fracture compared to the adults. No significant differences were found between the adolescents and adults regarding Injury Severity Score (ISS), New Injury Severity Score (NISS), Trauma-Injury Severity Score (TRISS), mortality, length of hospital stay, or intensive care unit (ICU) admission rate. A significantly greater percentage of adolescents compared to adults were found not to have worn a helmet. Motorcycle riders who had not worn a helmet were found to have a significantly lower first Glasgow Coma Scale (GCS) score, and a significantly higher percentage was found to present with unconscious status, head and neck injury, and cranial fracture compared to those who had worn a helmet.

**Conclusion:**

Adolescent motorcycle riders comprise a major population of patients hospitalized for treatment of trauma. This population tends to present with a higher injury severity compared to other hospitalized trauma patients and a bodily injury pattern differing from that of adult motorcycle riders, indicating the need to emphasize use of protective equipment, especially helmets, to reduce their rate and severity of injury.

## Background

Road traffic accidents have been reported as the most common causes of blunt pediatric injuries [1, 2]. Among the various means of transportation, motorcycle use is becoming popular in many cities as a cheaper, easier, and more fuel-efficient means. However, the increased use of motorcycles for recreation, the availability of more powerful motorcycles, and a greater number of older riders has led to increased incidence of motorcycle fatalities and injuries [3]. Motorcycle drivers are 35 times more likely than pillion-car occupants (i.e., motorcycle riders) to die in a motor vehicle traffic crash, 8 times more likely to be injured per vehicle mile [4], and 58 times more likely to be killed on a per-trip basis [5]. Among motorcycle drivers, young motorcyclists have the highest fatality rates of any age group, perhaps owing to their inexperience, skill level, and risky riding behavior [6]. In the United States, the national population estimate for all motorcycle-related hospital discharges for patients aged 12–20 years in 2006 was 5,662, a figure that represented 3.0 % of all hospitalized injuries for this age group [7]. The Centers for Disease Control and Prevention (CDC) reported that the motorcyclist fatality rate for individuals aged 12–20 had increased from 0.52 deaths per 100,000 population in 1999 to 0.98 deaths per 100,000 population in 2006, an increase of 88 % [7].

Pediatric patients sustain distinct patterns of injuries from causes that differ from those of adults because of their unique anatomical, physiologic, and behavioral characteristics. Young motorcyclists are considered a high-risk traffic group [8, 9] because they are more likely to be at fault in the event of a collision due to being under the influence of alcohol, riding without insurance, or not wearing a helmet [9]. While a significant link has been found between risk perception and traffic condition awareness for experienced drivers (ages 25–28), it has not been found for younger drivers (ages 18–24) [10].

The figures regarding the number of major trauma patients and the subsequent volume of surgery performed for those aged 10–17 years have been reported to differ from those reported for younger patients [3]. The identification of high-risk injury patterns may lead to improved care and ultimately further improvements in outcome in children and adolescents admitted to the hospital with trauma [11, 12]. In addition, gaining greater understanding of the epidemiology of pediatric major trauma is vital to integrate the knowledge of pediatric trauma into the trauma system to maximize the provision of services and quality of care delivered. To assist in achieving these aims, this study investigated the injury pattern, mechanisms, severity, and mortality of adolescents treated for injuries sustained in motorcycle accidents in a Level I trauma center in southern Taiwan using data from a population-based trauma registry.

## Methods

### Study design

The study was conducted at Kaohsiung Chang Gung Memorial Hospital, a 2,400-bed facility and a Level I regional trauma center that provides care to trauma patients primarily from South Taiwan. Approval for this study was obtained by the hospital institutional review board (approval number 103-2186B) before its initiation. An informed consent was waived according to the regulation of IRB. This retrospective study was designed to review all the data added to the Trauma Registry System from January 1, 2009 to December 31, 2012 for selection of cases that met the inclusion criteria of (1) age 13–19 years (adolescents) or age 30–50 years (adults) and (2) hospitalization for treatment of trauma following a motorcycle accident. The lower age limit for adolescents was chosen because of the recent sharp increase in incidence observed for cases as young as age 13. Exclusion criteria included those patients with incomplete data. The aim of selection of this age group (age 13–19) rather than a younger or older group was to narrow the selected range of ages to avoid comparison with those just older than 20 years and to avoid the introduction of the possibly confounding factor of inability to control a motorcycle due to advanced age, a factor generally affecting those over 60 years. To compare the injury pattern, mechanisms, severity, and mortality of adolescents from those of adults hospitalized for treatment of trauma following motorcycle accidents, the data of patients who had sustained injuries in a motorcycle accident, including road and off-road motorcyclist accidents, were collected for further analysis.

Among the 13,233 hospitalized registered patients entered in the database, 1,033 (7.8 %) were adolescents ages from 13 to 19 years and 3,470 (26.2 %) adults between 30 and 50 years. Among them, 635 (61.5 %) adolescents and 1,566 (45.1 %) adults had been admitted due to a motorcycle accident. Detailed patient information was retrieved from the Trauma Registry System of our institution and included data regarding age, sex, admission vital signs, injury mechanism, helmet use, the first Glasgow Coma Scale (GCS) in the emergency department, Abbreviated Injury Scale (AIS) severity score of each body region, Injury Severity Score (ISS), New Injury Severity Score (NISS), Trauma-Injury Severity Score (TRISS), length of hospital stay (LOS), length of intensive care unit stay (LICUS), in-hospital mortality, and rates of associated complications. Odd ratios (ORs) of the associated injuries of adolescents and adults in the motorcycle accidents were calculated with 95 % confidence intervals (CIs). The data collected regarding the combined population of drivers and pillions (hereafter referred to as riders) were compared using SPSS v.20 statistical software (IBM, Armonk, NY, USA) for performance of Pearson’s chi-squared test, Fisher’s exact test, or the independent Student’s t-test, as applicable. All results are presented as the mean ± standard error. A p-value less than 0.05 was considered statistically significant.

## Results

### Characteristics of all trauma patients

The mean age was 16.9 ± 1.9 and 40.5 ± 6.1 years, respectively, in the adolescent and adult patient groups (Table 1). Of the 1,033 adolescents, 737 (71.3 %) were male and 296 (28.7 %), female. Of the 3,470 adult patients, 2,438 (70.3 %) were male and 1,032 (29.7 %) female. No statistically significant difference was found between the groups regarding sex. Among the injured patients, 2053 (547 [53.0 %] of the adolescents and 1506 [43.4 %] of the adults) were the drivers of motorcycles and only 148 (88 [8.5 %] of the adolescents and 60 [1.7 %] of the adults) were the riders.

**Table 1:** Demographics of hospitalized trauma patients aged 13–19 years (adolescents) and 30–50 years (adults)

Variable	Adolescent	Adult	*p*
	*N* = 1033	*N* = 3470	
Age	16.9 ± 1.9	40.5 ± 6.1	
Gender, n(%)			0.502
Male	737(71.3)	2438(70.3)	
Female	296(28.7)	1032(29.7)	
Mechanism, n(%)			
Drivers of MV	2(0.2)	96(2.8)	
Pillions of MV	14(1.4)	35(1.0)	
Drivers of Motorcycle	547(53.0)	1506(43.4)	
Pillions of Motorcycle	88(8.5)	60(1.7)	
Bicyclists	67(6.5)	89(2.6)	
Pedestrians	14(1.4)	44(1.3)	
Fall	140(13.6)	570(16.4)	
Unspecific	161(15.6)	1070(30.8)	
ISS	7.8 ± 7.0	7.7 ± 6.7	0.571
< 16	903(87.4)	3041(87.6)	0.850
16-24	89(8.6)	318(9.2)	0.590
≥ 25	41(4.0)	111(3.2)	0.229
NISS	8.9 ± 7.8	8.7 ± 7.7	0.460
TRISS	0.976 ± 0.099	0.979 ± 0.093	0.451
Mortality, n(%)	4(0.39)	33(0.95)	0.078

### Injury severity of all trauma patients

Comparison of trauma injury scores for the adolescent and adult groups did not indicate any significant difference regarding ISS (7.8 ± 7.0 vs. 7.7 ± 6.7, respectively, *p* = 0.571) for any subgroup of injury severity (ISS <16, 16–24, and ≥25) or regarding NISS (8.9 ± 7.8 vs. 8.7 ± 7.7, respectively, *p* = 0.460), TRISS (0.976 ± 0.099 vs. 0.979 ± 0.093, respectively, *p* = 0.451), or in-hospital mortality (0.39 % vs. 0.95 %, respectively, *p* = 0.078). In contrast, a significant difference in ISS was found between the 635 adolescent riders (ISS = 9.2 ± 7.6) and the other 398 adolescent non-motorcycle riders (*p* < 0.001).

### Characteristics of the motorcycle-related trauma patients

**Fig. 1:**
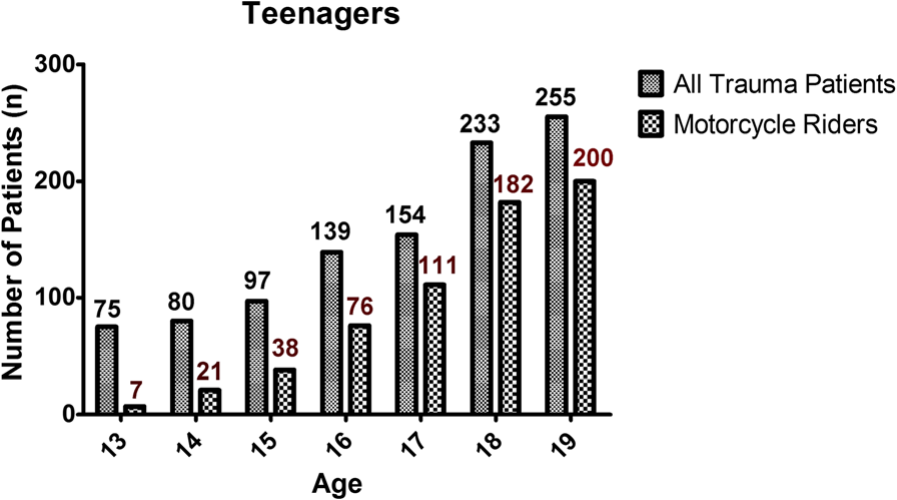
Number of adolescent patients admitted for treatment of all trauma injury and number admitted for treatment of motorcycle-related trauma injury

The data regarding the 635 (61.5 %) adolescent and 1566 (45.1 %) adult patients who had been motorcycle riders were further compared for identification of differences regarding motorcycle-related major trauma injury. As shown in Fig. 1, of the 75, 80, 97, 139, 154, 233, and 255 hospitalized patients aged 13, 14, 15, 16, 17, 18, and 19 years, respectively, 7 (9.3 %), 21 (26.3 %), 38 (39.2 %), 76 (54.7 %), 111 (72.1 %), 182 (78.1 %), and 200 (78.4 %) patients, respectively, had been admitted for treatment subsequent to a motorcycle accident. Among these adolescent motorcycle riders, 77.6 % (*n* = 493) were aged from 17 to 19 years. As shown in Table 2, of the 635 adolescent and 1566 adult motorcycle riders, the mean age was 17.5 ± 1.4 and 40.1 ± 6.2 years, respectively. No statistically significant difference was found regarding sex between the adolescent motorcycle riders, of whom 416 (65.5 %) were male and 219 (34.5 %) female, and the adult motorcycle riders, of whom 988 (63.1 %) were male and 578 (36.9 %) female. Analysis of the data regarding helmet-wearing status, which were recorded for 97.5 % of the adolescent and 97.3 % of the adult patients, revealed that significantly more adolescent motorcycle drivers had not been wearing a helmet compared to the adult motorcycle drivers (12.4 % vs. 10.0 %, respectively, *p* = 0.012). In contrast, no significant difference regarding helmet-wearing status was found between the adolescent and adult motorcycle pillions. In addition, 455 of the 534 adolescent drivers and 67 of the 85 adolescent pillions had worn a helmet (*p* = 0.133) and 1307 of the 1464 adult drivers and 53 of the 59 adult pillions had worn a helmet (*p* = 0. 0.893). No significant differences regarding helmet wearing was found between drivers and pillions in either group.

**Table 2:** Injury characteristics of adolescent and adult motorcycle riders

Motorcycle Accident			
	Adolescent	Adult	*p*
	*N* = 635	*N* = 1566	
Age	17.5 ± 1.4	40.1 ± 6.2	
Gender, n(%)			0.284
Male	416(65.5)	988(63.1)	
Female	219(34.5)	578(36.9)	
Helmet wearing, n(%)			
Yes			
Drivers	455(71.7)	1307(83.5)	0.038
Pillions	67(10.6)	53(3.4)	0.063
No			
Drivers	79(12.4)	157(10.0)	0.012
Pillions	18(2.8)	6(0.4)	0.090
Unknown	16(2.5)	43(2.7)	0.766
GCS	14.2 ± 2.3	14.1 ± 2.6	0.457
≤ 8	30(4.7)	85(5.4)	0.502
9–12	37(5.8)	98(6.3)	0.702
≥ 13	568(89.4)	1383(88.3)	0.447
AIS n(%)			
Head/Neck	224(35.3)	521(33.3)	0.368
Face	194(30.6)	403(25.7)	0.021
Thorax	54(8.5)	250(16.0)	0.000
Abdomen	65(10.2)	111(7.1)	0.014
Extremity	442(69.6)	1183(75.5)	0.004
ISS	9.2 ± 7.6	9.2 ± 7.2	0.914
< 16	525(82.7)	1301(83.1)	0.821
16–24	79(12.4)	195(12.4)	0.994
≥ 25	31(4.9)	70(4.5)	0.6766
NISS	10.5 ± 8.4	10.5 ± 8.2	0.945
TRISS	0.971 ± 0.110	0.975 ± 0.095	0.386
Mortality, n(%)	3(0.5 %)	20(1.3 %)	0.093
LOS (days)	9.5 ± 9.5	9.1 ± 9.3	0.460
ICU			
Patients, n(%)	125(19.7)	262(16.7)	0.099
< 16	45(8.6)	91(7.0)	0.245
16–24	55(69.6)	114(58.5)	0.085
≥ 25	25(80.6)	57(81.4)	0.926
LOS in ICU (days)	6.4 ± 6.2	6.4 ± 6.3.	0.791
< 16	5.67.1	4.8 ± 5.2	0.207
16–24	6.6 ± 5.7	5.6 ± 4.7	0.061
≥ 25	7.3 ± 5.6	10.5 ± 8.7	0.068

### Injury severity of the motorcycle-related trauma patients

No significant difference was found between the adolescent and adult patients regarding GCS score (14.2 ± 2.3 vs.14.1 ± 2.6, respectively, *p* = 0.457) or distribution of patients at different levels of consciousness (GCS ≤8, 9–12, or ≥13; Table 3). Moreover, no significant differences in GCS score was found between the adolescent drivers (*n* = 547, 14.2 ± 2.4) and the adolescent pillions (*n* = 88, 14.1 ± 2.6; *p* = 0.737) or between the adult drivers (*n* = 1506, 14.1 ± 2.6) and the adult pillions (*n =* 60, 14.0 ± 3.0; *p* = 0.788). Likewise, no significant differences were found between the adolescent and adult motorcycle riders regarding ISS (9.2 ± 7.6 vs. 9.2 ± 7.2, respectively, *p* = 0.914) regardless of subgroup of injury severity; NISS (10.5 ± 8.4 vs. 10.5 ± 8.2, respectively, *p* = 0.945); TRISS (0.971 ± 0.110 vs. 0.975 ± 0.095, respectively, *p* = 0.386); or in-hospital mortality (0.5 % vs. 1.3 %, respectively, *p* = 0.093). Moreover, no significant differences regarding hospital LOS (9.5 days vs. 9.1 days, respectively, *p* = 0.460), proportion of patients admitted to the intensive care unit (ICU; 19.7 % vs. 16.7 %, respectively, *p* = 0.099), or LICUS (6.4 days vs. 6.4 days, respectively, *p* = 0.791), regardless of injury severity, were found between the adolescent and adult motorcycle riders.

**Table 3:** Associated injuries of adolescent and adult motorcycle riders

Motorcycle accident				
	Adolescent *N* = 635	Adult *N* = 1566	*Odds Ratio (95%CI)*	*p*
Head trauma, n(%)				
Neurologic deficit	6(0.9)	20(1.3)	0.7(0.30–1.85)	0.513
Cranial fracture^a^	89(14.0)	146(9.3)	1.6(1.20–2.10)	0.001
Epidural hematoma (EDH)	13(2.0)	24(1.5)	1.3(0.68–2.65)	0.395
Subdural hematoma (SDH)	34(5.4)	79(5.0)	1.1(0.71–1.61)	0.766
Subarachnoid hemorrhage (SAH)	40(6.3)	134(8.6)	0.7(0.50–1.04)	0.075
Intracerebral hematoma (ICH)	12(1.9)	25(1.6)	1.2(0.59–2.38)	0.628
Cerebral contusion	29(4.6)	72(4.6)	1.0(0.64–1.54)	0.975
Cervical vertebral fracture	2(0.3)	14(0.9)	0.4(0.08–1.55)	0.147
Maxillofacial trauma, n(%)				
Maxillary fracture	65(10.2)	182(11.6)	0.9(0.64–1.17)	0.351
Mandibular fracture^a^	47(7.4)	52(3.3)	2.3(1.55–3.49)	0.000
Orbital fracture	28(4.4)	54(3.4)	1.3(0.81–2.06)	0.281
Nasal fracture	13(2.0)	29(1.9)	1.1(0.57–2.15)	0.761
Thoracic trauma, n(%)				
Rib fracture^+^	9(1.4)	183(11.7)	0.1(0.06–0.21)	0.000
Hemothorax^+^	5(0.8)	39(2.5)	0.3(0.12–0.79)	0.010
Pneumothorax	17(2.7)	30(1.9)	1.4(0.77–2.57)	0.263
Lung contusion	7(1.1)	24(1.5)	0.7(0.31–1.67)	0.438
Hemopneumothorax	9(1.4)	26(1.7)	0.9(0.40–1.83)	0.680
Thoracic vertebral fracture	1(0.2)	15(1.0)	0.2(0.02–1.24)	0.053
Abdominal trauma, n(%)				
Intra-abdominal injury	21(3.3)	33(2.1)	1.6(0.91–2.77)	0.099
Hepatic injury^a^	32(5.0)	32(2.0)	2.5(1.55–4.19)	0.000
Splenic injury	13(2.0)	29(1.9)	1.1(0.57–2.15)	0.761
Retroperitoneal injury	1(0.2)	4(0.3)	0.6(0.07–5.52)	0.662
Renal injury	6(0.9)	7(0.4)	2.1(0.71–6.35)	0.167
Urinary bladder injury	2(0.3)	4(0.3)	1.2(0.23–6.75)	0.808
Lumbar vertebral fracture	4(0.6)	24(1.5)	0.4(0.14–1.18)	0.087
Sacral vertebral fracture	4(0.6)	12(0.8)	0.8(0.26–2.56)	0.733
Extremity trauma, n(%)				
Scapular fracture^+^	7(1.1)	47(3.0)	0.4(0.16–0.80)	0.009
Clavicle fracture^+^	41(6.5)	273(17.4)	0.3(0.23–0.46)	0.000
Humeral fracture^+^	19(3.0)	86(5.5)	0.5(0.32–0.88)	0.013
Radial fracture	59(9.3)	150(9.6)	1.0(0.71–1.33)	0.835
Ulnar fracture	26(4.1)	68(4.3)	0.9(0.59–1.49)	0.794
Femoral fracture^a^	107(16.9)	126(8.0)	2.3(1.76–3.05)	0.000
Patella fracture	21(3.3)	45(2.9)	1.2(0.68–1.96)	0.589
Tibia fracture	60(9.4)	117(7.5)	1.3(0.93–1.79)	0.122
Fibular fracture	43(6.8)	94(6.0)	1.1(0.78–1.65)	0.499
Metacarpal fracture	24(3.8)	50(3.2)	1.2(0.73–1.96)	0.489
Metatarsal fracture	23(3.6)	38(2.4)	1.5(0.89–2.56)	0.122
Calcaneal fracture	27(4.3)	99(6.3)	0.7(0.43–1.02)	0.058
Pelvic fracture	20(3.1)	49(3.1)	1.0(0.59–1.71)	0.980

^+^ and ^a^ indicated significant lower and higher incidences of the associated injury, respectively, in the adolescents than those adult patients (*p*<0.05).

### Injury pattern of the motorcycle-related trauma patients

Analysis of AIS revealed that the adolescent patients had sustained significantly higher rates of facial injury (30.6 % vs. 25.7 %, respectively, *p* = 0.021) and abdominal injury (10.2 % vs. 7.1 %, respectively, *p* = 0.014) compared to the adult patients, while the adult patients had sustained significantly higher rates of thorax injury (16.0 % vs. 8.5 %, respectively, *p* = 0.000) and extremity injury (75.5 % vs. 69.6 %, respectively, *p* = 0.004). On the other hand, no significant differences regarding injury to the head and neck region were found between the adolescent and adult patients. Table 3 shows the findings regarding injury associated with motorcycle accidents. As can be observed, a significantly higher percentage of adolescent riders had sustained cranial fracture (OR = 1.6, 95 % CI = 1.20–2.10), mandibular fracture (OR = 2.3, 95 % CI = 1.55–3.49), hepatic injury (OR = 2.5, 95 % CI = 1.55–4.19), or femoral fracture (OR = 2.3, 95 % CI: 1.76–3.05) compared to adult riders. In contrast, a significantly lower percentage of adolescent motorcycle riders had sustained rib fracture (OR = 0.1, 95 % CI = 0.06–0.21), hemothorax (OR = 0.3, 95 % CI = 0.12–0.79), scapular fracture (OR = 0.4, 95 % CI = 0.16–0.80), clavicle fracture (OR = 0.3, 95 % CI = 0.23–0.46), and humeral fracture (OR = 0.5, 95 % CI = 0.32–0.88).

### Helmet-wearing status of the motorcycle-related trauma patients

Table 4 shows the results of the analysis of helmet-wearing status among adolescent riders. As can be observed, adolescent riders who had not worn a helmet presented with a significantly lower first GCS score compared to those who had worn a helmet (13.1 ± 2.9 vs. 14.4 ± 2.1, respectively, *p* = 0.000). A significantly greater percentage of adolescent riders who had not worn a helmet presented with unconscious status based on GCS score ≤8 (12.4 % vs. 4.4 %, respectively, *p* = 0.002), head and neck injury based on AIS (52.6 % vs. 30.8 %, respectively, *p* = 0.000), and cranial fracture (26.8 % vs. 10.9 %, respectively, *p* = 0.000), while a significantly lower percentage presented with extremity injury based on AIS (59.8 % vs. 72.2 %, respectively, *p* = 0.014). In contrast, no significant differences were found between adolescent riders who had and had not worn a helmet regarding incidence of maxillofacial trauma, regardless of the type of maxillofacial trauma. While significantly more patients who had not worn a helmet had sustained severe injury (ISS 16–24; 18.6 % vs. 10.5 %, respectively, *p* = 0.025), significantly fewer patients who had not worn a helmet had an ISS less than 16 (76.3 % vs. 85.2 %, respectively, *p* = 0.028). Although a significantly higher percentage of adolescents who had not worn a helmet required admission to the ICU (32.0 % vs. 16.7 %, respectively, *p* = 0.000), no significant differences were found between adolescents who had and had not worn a helmet regarding incidence of very severe injury (ISS ≥ 25), NISS, TRISS, mortality, LOS, or LICUS.

**Table 4:** Injury characteristics of adolescent motorcycle riders according to helmet-wearing status

Motorcycle accident			
	Helmet + *N* = 522	Helmet-*N* = 97	*p*
Gender, n(%)			0.972
Male	340(65.1)	63(64.9)	
Female	182(34.9)	34(35.1)	
GCS	14.4 ± 2.1	13.1 ± 2.9	0.000
≤ 8	23(4.4)	12(12.4)	0.002
9–12	22(4.2)	2(2.1)	0.313
≥ 13	477(91.4)	83(85.6)	0.073
AIS n(%)			
Head/Neck	161 (30.8)	51 (52.6)	0.000
Face	156 (29.9)	33 (34.0)	0.417
Thorax	46 (8.8)	6 (6.2)	0.392
Abdomen	54 (10.3)	9 (9.3)	0.750
Extremity	377 (72.2)	58 (59.8)	0.014
Head trauma, n(%)			
Neurologic deficit	4(0.8)	1(1.0)	0.789
Cranial fracture^a^	57(10.9)	26(26.8)	0.000
Epidural hematoma (EDH)	9(1.7)	3(3.1)	0.369
Subdural hematoma (SDH)	20(3.8)	8(8.2)	0.055
Subarachnoid hemorrhage (SAH)	30(5.7)	7(7.2)	0.575
Intracerebral hematoma (ICH)	9(1.7)	1(1.0)	0.619
Cerebral contusion	21(4.0)	3(3.1)	0.663
Cervical vertebral fracture	2(0.4)	0(0.0)	0.541
Maxillofacial trauma, n(%)			
Maxillary fracture	52(10.0)	10(10.3)	0.917
Mandibular fracture	38(7.3)	7(7.2)	0.982
Orbital fracture	25(4.8)	3(3.1)	0.460
Nasal fracture	10(1.9)	3(3.1)	0.458
ISS	8.9 ± 7.5	9.8 ± 7.2	0.383
< 16	445(85.2)	74(76.3)	0.028
16–24	55(10.5)	18(18.6)	0.025
≥ 25	22(4.2)	5(5.2)	0.677
NISS	10.2 ± 8.3	11.0 ± 8.0	0.455
TRISS	0.973 ± 0.110	0.971 ± 0.106	0.997
Mortality, n(%)	2(0.4 %)	0(0.0 %)	0.541
LOS (days)	9.1 ± 8.7	8.7 ± 7.0	0.077
ICU			
Patients, n(%)	87(16.7)	31(32.0)	0.000
LICUS (days)	6.3 ± 6.4	5.9 ± 5.5	0.612

## Discussion

This study analyzed the demographics and characteristics of injuries observed in a population of adolescents with motorcycle-related injuries presenting at a Level I trauma center. Analysis of the data indicates that adolescent motorcycle riders comprise a major population of hospitalized trauma patients, have a higher severe injury score compared to adolescents hospitalized for all trauma injury, and present with a bodily injury pattern that differs from that of adult motorcycle riders. It also revealed that a significant percentage of adolescent motorcycle riders do not wear a helmet, which, as motorcyclists have little other protection from injury, puts them at high risk of injury.

A previous study found that the youngest motorcyclists, defined as those aged 16–19 years, were 1.30 (95 % CI = 1.10–1.54), 3.09 (95 % CI = 2.61–3.66), and 4.79 (95 % CI = 4.04–5.67) times more likely to be killed and 3.67 (95 % CI = 3.34–4.03), 10.68 (95 % CI = 9.73–11.71), and 18.03 (95 % CI = 16.43–19.78) times more likely to be nonfatally injured compared to motorcyclists aged 20–29, 30–39, and 40–49 years, respectively [9]. In the current study, no significant differences were found between adolescent and adult motorcycle riders regarding ISS, regardless of the subgroup of injury severity; NISS, TRISS; mortality; hospital LOS; proportion admitted to the ICU; or LICUS. Similar studies in Singapore also reported that most motorcyclist riders hospitalized for treatment of trauma had a low ISS [13]. Considering that almost all of motorcycles are forbidden on highways in Asian cities and that most traffic accidents occur in relatively crowded streets in these cities, we hypothesize that the reason for the discrepancy between our findings and those of previous Western studies is that most motorcycle injuries in the Asian region occur at relatively low velocity.

In contrast, the adolescent motorcycle riders were found to have presented with a different bodily injury pattern compared to the adult motorcycle riders. Based on analysis of AIS, the adolescent riders presented with a higher rate of injury to the face and abdomen, but a lower rate to the thorax and extremities, and a higher rate of cranial, mandibular, hepatic, and femoral fracture but a lower rate of hemothorax and rib, thoracic vertebral, scapular, clavicle, and humeral fracture. Notably, the adolescent motorcycle riders sustained a more than 2-fold greater incidence of mandibular fracture, hepatic injury, and femoral fracture compared to the adult motorcycle riders, whereas the latter sustained a significantly higher rate of injury around the thorax region; rib fracture, hemothorax, and scapular, clavicle, and humeral fracture, all of which are considered within the category of extremity injury.

A previous study by Jou et al. in Taiwan revealed that motorcyclist fatality accounted for nearly 60 % of all driving fatalities in the country between 2006 and 2008 [14]. They also found an association between higher fatality rates and the factors of male sex, advanced age, unlicensed status, not wearing a helmet, riding after alcohol consumption, and alcohol consumption of more than 550 cc [14]. In the current study, 3 of 4 (75 %) fatalities among adolescents aged 13–19 years and 20 of 33 (61 %) among adults aged 30–50 years were found to have involved motorcycle use. However, the number of fatalities among the adolescent motorcycle riders examined was too small to analyze and draw any conclusions from.

Among several preventive measures, helmet wearing in particular has been shown to protect against head and other serious injuries and to be cost effective [9, 15, 16]. One study found a 37 % increased risk of serious/severe traumatic brain injury that required hospitalization for young motorcycle riders in states with limited-age helmet laws compared with youth in states with universal helmet laws, with the greatest increase in risk observed for the most severe type of head injury in the largest group of injured young motorcycle riders: those aged 18–20 [4]. The study also revealed that the decrease in helmet-usage rates for youth when universal helmet laws are repealed leads to increases youth motorcycle fatality rates and overall morbidity [4]. In the current study, adolescent motorcycle drivers, but not pillions, were found to be less likely to wear a helmet than adult motorcycle drivers. Compared to patients who had worn a helmet, a greater number of patients who had not worn a helmet presented with unconscious status (GCS score ≤8); had sustained head and neck injury, cranial fracture, and severe injury (ISS 16–24); and had required admission to the ICU. These findings indicate that wearing a helmet may prevent head injury and reduce injury severity among adolescent motorcycle riders. However, among adolescent motorcycle riders who had sustained very severe injury (ISS ≥25), no significant difference was found regarding the percentage who had and had not worn a helmet. Moreover, no significant differences regarding NISS, TRISS, mortality, LOS, or LICUS were found between those adolescent motorcycle riders who had and had not worn a helmet.

The limitations of this study include the use of a retrospective design and the lack of availability of data regarding the circumstances of the mechanism of injury. Although a study regarding the factors influencing motorcycle crash victim outcomes found that traveling in excess of 50 kph increased the risk of intracranial injury (OR = 4.8) [17], lack of data regarding the motorcycle speed during accidents prevented analysis of the effect of speed in the current study. Lack of data also prevented the ability to analyze the impact of the type of motorcycle; type of helmet material; or the use of any other protective materials, such as knee braces. Lastly, lack of exposure data prevented analysis of motorcycle-related hospitalization based on exposure-based risk (e.g., number of trips, hours of riding, and/or miles traveled). As younger motorcycle riders generally do not own motorcycles or travel as much as their older counterparts in terms of distance and time, inability to analyze exposure data may have led to underestimation of the true risk for younger age groups.

## Conclusion

Adolescent motorcycle riders comprise a major population of patients hospitalized for treatment of trauma. This population tends to present with a higher injury severity compared to other trauma patients and a bodily injury pattern differing from that of adult motorcycle riders, indicating the need to emphasize the use of protective equipment, especially helmets, to reduce their rate and severity of injury.
